# Profiling of m6A RNA modifications identified an age‐associated regulation of *AGO2* mRNA stability

**DOI:** 10.1111/acel.12753

**Published:** 2018-03-23

**Authors:** Kyung‐Won Min, Richard W. Zealy, Sylvia Davila, Mikhail Fomin, James C. Cummings, Daniel Makowsky, Catherine H. Mcdowell, Haley Thigpen, Markus Hafner, Sang‐Ho Kwon, Constantin Georgescu, Jonathan D. Wren, Je‐Hyun Yoon

**Affiliations:** ^1^ Department of Biochemistry and Molecular Biology Medical University of South Carolina Charleston SC USA; ^2^ Laboratory of Muscle Stem Cells and Gene Regulation National Institute of Arthritis and Musculoskeletal and Skin Diseases Bethesda MD USA; ^3^ Department of Medicine Division of Nephrology Medical University of South Carolina Charleston SC USA; ^4^ Arthritis and Clinical Immunology Research Program Division of Genomics and Data Sciences Oklahoma Medical Research Foundation Oklahoma City OK USA; ^5^ Laboratory of Genetics National Institute on Aging‐Intramural Research Program, NIH Baltimore MD USA

**Keywords:** aging, m6A RNA methylation, post transcriptional gene regulation

## Abstract

Gene expression is dynamically regulated in a variety of mammalian physiologies. During mammalian aging, there are changes that occur in protein expression that are highly controlled by the regulatory steps in transcription, post‐transcription, and post‐translation. Although there are global profiles of human transcripts during the aging processes available, the mechanism(s) by which transcripts are differentially expressed between young and old cohorts remains unclear. Here, we report on N6‐methyladenosine (m6A) RNA modification profiles of human peripheral blood mononuclear cells (PBMCs) from young and old cohorts. An m6A RNA profile identified a decrease in overall RNA methylation during the aging process as well as the predominant modification on proteincoding mRNAs. The m6A‐modified transcripts tend to be more highly expressed than nonmodified ones. Among the many methylated mRNAs, those of *DROSHA* and *AGO2* were heavily methylated in young PBMCs which coincided with a decreased steady‐state level of *AGO2* mRNA in the old PBMC cohort. Similarly, downregulation of AGO2 in proliferating human diploid fibroblasts (HDFs) also correlated with a decrease in *AGO2* mRNA modifications and steady‐state levels. In addition, the overexpression of RNA methyltransferases stabilized *AGO2* mRNA but not *DROSHA* and *DICER1* mRNA in HDFs. Moreover, the abundance of miRNAs also changed in the young and old PBMCs which are possibly due to a correlation with AGO2 expression as observed in AGO2‐depleted HDFs. Taken together, we uncovered the role of mRNA methylation on the abundance of *AGO2* mRNA resulting in the repression of miRNA expression during the process of human aging.

## 1 INTRODUCTION

Toward the goal of increasing human lifespan of humans, there is great interest in identifying the underlying molecular causes behind the age‐associated loss of physiological functions and rise in pathologies. Recent studies using high‐throughput RNA sequencing technology have revealed that the expression of mammalian mRNAs changes extensively during the aging process (Baumgart et al., 2016; White et al., 2015). The dynamic expression of mRNAs is mainly governed by transcription and decay via their interactions with trans‐regulatory factors (e.g., RNA‐binding proteins and noncoding RNAs) and cis‐regulatory elements (specific sequences on mRNAs for RNA‐binding protein interaction, miRNA targeting, RNA modification, etc.) (Grosswendt et al., 2014; Wang & He, 2014; Yoon, Abdelmohsen & Gorospe, 2013; Yoon et al., 2014).

Regulatory RNA modifications include A‐to‐I editing, pseudouridylation, methylation, and nicotinamide‐adenine dinucleotide (NAD^+^) capping, which can regulate many cellular processes by influencing RNA metabolism (Jiao et al., 2017; Nishikura, 2016; Walters et al., 2016; Wang et al., 2014; Zhao, Roundtree & He, 2017). For example, A‐to‐I editing within a coding region of mRNA may lead to abnormal protein expressions that are not encoded in the genome, and editing within intronic regions could create new splice sites resulting in the inclusion of an undesired sequence in the mature mRNA (Nishikura, 2016). Also, hundreds of pseudouridylated mRNAs were found in mammalian cells and, in response to serum starvation, dynamic changes of pseudouridylation were observed, underscoring its potential as a regulatory element (Carlile et al., 2014). As for RNA methylation, five types of methylation have been identified including *N* ^7^‐methylguanine (m^7^G at the 5' cap), *N* ^6^‐methyl adenosine (m^6^A), *N* ^1^‐methyl adenosine (m^1^A), 5‐methylcytosine (m^5^C), and 2‐*O*‐methylation (2'OME). Additionally, di‐methylation occurs when a combination of two different methylations affect the same mRNA, such as when m^6^Am is observed next to the 5' cap (Mauer et al., 2016).

Methylation profiles have revealed differential patterns of RNA modification, for instance, the pattern of m1A's are nearby translation start codons and first splice sites, and m6A sites are primarily within the 3' UTR (Dominissini et al., 2012, 2016). Recently, a study observed NAD^+^‐modified RNA has been observed in yeast and mammals, and their results implied a function in mRNA translation and decay (Jiao et al., 2017; Walters et al., 2016). These observations of differential RNA modifications have now opened a new area of research, “epitranscriptomics,” where the goal is to decode the fate of RNA modifications that impact decay, translation, and localization. Ultimately, post‐transcriptional RNA modification could shape cellular physiology and processes such as cell division, survival, stress response, and differentiation, which may impact disease progression, if, for example, cells cannot properly mark RNAs in order to regulate gene expression correctly in a given context.

Previously we identified RNAs that were differentially expressed in proliferating and senescent human diploid fibroblasts and found that many transcripts were overexpressed or underexpressed in senescent fibroblasts (Abdelmohsen et al., 2013). Given the relevance of cellular senescence to mammalian aging, we initiated a systemic investigation as to whether RNA modification regulates RNA abundance during human aging. Specifically, we focused on the m6A RNA modification because it is the most abundant internal modification observed in eukaryotic mRNA, which can affect mRNA splicing and nuclear export; in the cytoplasm, m6A mRNAs are recognized by the RNA‐binding protein (RBP) that will mobilize them to processing (P) bodies, where mRNA stability and translation are modulated (Fu, Dominissini, Rechavi & He, 2014; Licht & Jantsch, 2016; Tang et al., 2015).

Here, we profile m6A modification of the PBMCs RNA, comparing young versus old donors to identify differences in mRNA expression and miRNA abundance, thus implicating their involvement in the aging and senescence processes. We identified that global m6A modification mainly occurs in mRNAs and the expression level of a subset of methylated mRNAs decreases in old cohorts. Specifically, *AGO2* mRNA is highly methylated in young PBMCs with a concomitant decrease in its stability in old cohorts. A similar change was observed in proliferating and senescent fibroblasts. Global changes in the expression of miRNA also suggest that *AGO2* mRNA methylation, its stability, and protein levels in old cohorts may be a factor to regulate miRNA abundance and stability.

## 2 RESULTS

### 2.1 m6A RNA sequencing in young and old PBMCs

**Figure 1:**
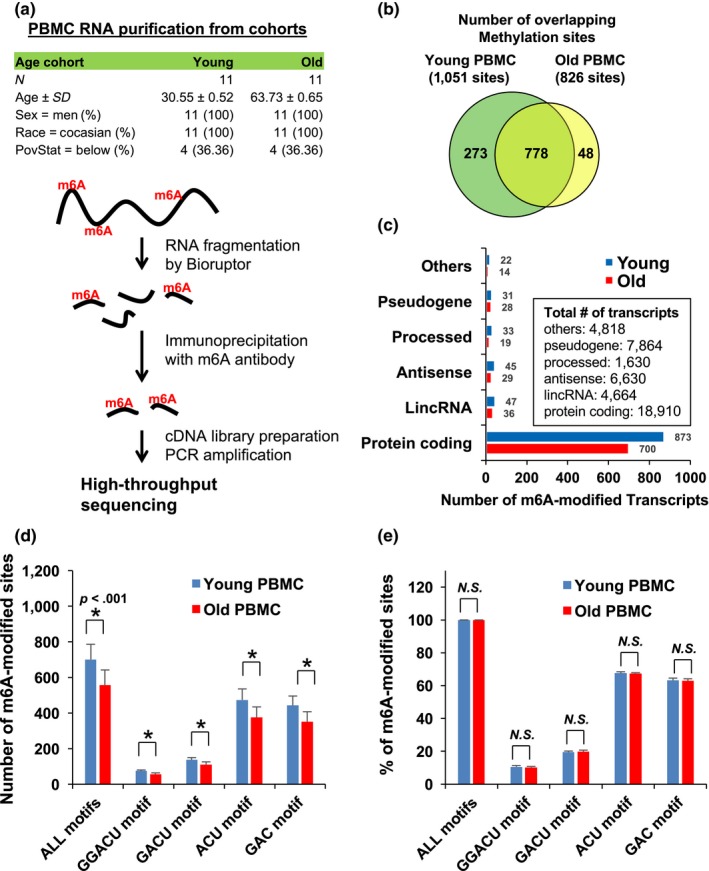
m6A RNA sequencing in young and old PBMCs. (a) Schematic of m6A RNA sequencing with PBMCs from young and old cohorts (b) Number of overlapping m6A sites on young and old PBMC RNAs (c) Number of RNA methylation sites on each transcripts types (d and e) Number *(D)* and proportions *(E)* of m6A RNAs in young and old PBMCs. Data in D are statistically significant using Student's *t*‐test (*p *=* *.000044, .000015, .000028, .000083, or .000067, respectively)

To better understand the roles of m6A RNA modification during the aging process, we set up an antibody‐based method that recognizes m6A. With which we immunoprecipitated (IP) m6A‐modified RNA collected from 11 young and 11 old individuals (Figure 1a) and then used high‐throughput RNA sequencing (MeRIP‐seq). Peripheral blood mononuclear cells (PBMCs) were donated by Caucasian males with the mean age of the “young” groups being 30.55(±0.52) and, for the “old” group, 63.73 (±0.65). We purified total RNAs from the PBMCs, physically fragmented them, generated cDNA libraries, and sequenced them as detailed in the Methods section.

Between the young and old groups, our analysis identified 1,051 methylated (m6A modification) fragments in the young PBMCs and 826 in the old PBMCs with 778 overlapping sites (Figure 1b and Table S1). m6A RNA predominantly occurs in protein‐coding RNAs found in both the young and old PBMCs (Figure 1c), which support earlier observations that m6A methylation occurs primarily in protein‐coding transcripts (Dominissini et al., 2012; Meyer Kate et al., 2012). However, methylation events occur at a low rate, less than 1% of the total number of transcripts. To quantitatively analyze m6A modification, we divided the transcripts by their methylation motifs (GGACU, GACU, ACU, or GAC), which revealed that overall methylation decreased in the old PBMCs (Figure 1d), while the relative proportion of each methylated motif remained constant between the two age groups (Figure 1e).

**Figure 2:**
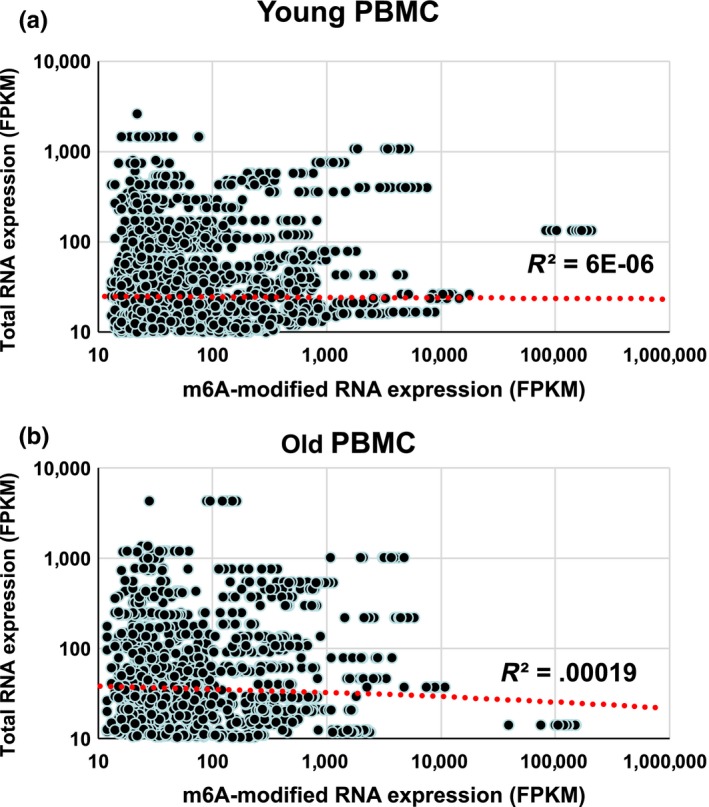
General relationship between mRNA expression and m6A methylation level. (a, b) Comparison of number of m6A methylation with total RNA expression in the same cohort group. Scatter plot comparing total RNA expression (FPKM) on the y‐axis with the number of m6A‐modified RNA expression (FPKM) on the x‐axis in the sample cohort group

**Figure 3:**
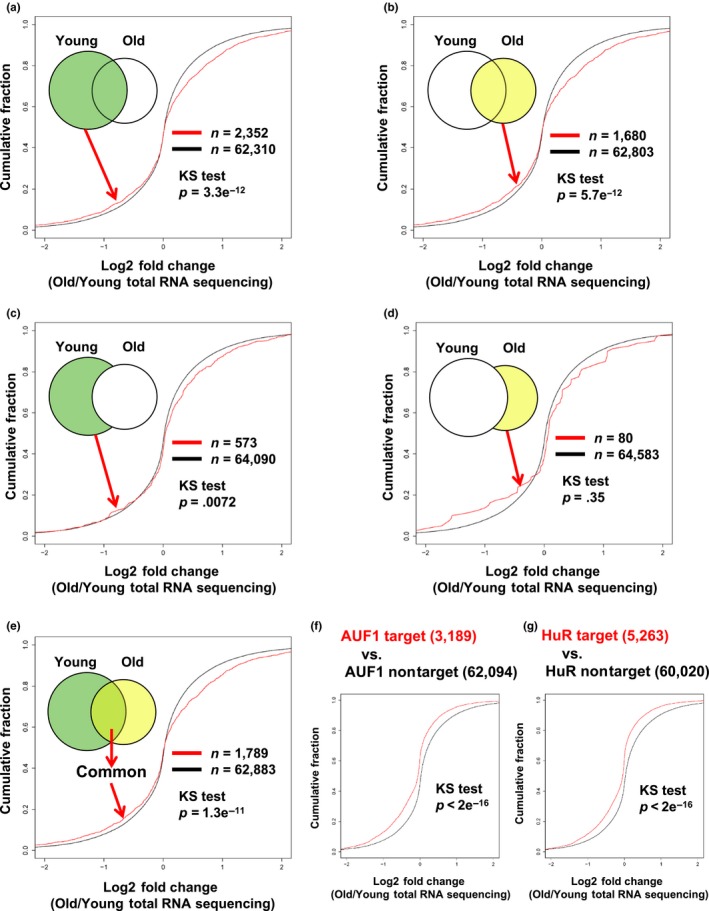
Cumulative Distribution Fraction Analysis of m6A‐methylation and steady‐state mRNA expression. (a–d) Cumulative distribution function plots showing the expression of m6A‐modified RNA compared to rest of RNA. The log2 fold changes in m6A‐modified RNA expression from old group over young group. (f, g) Cumulative distribution function plot showing the expression level of methylated AUF1 target and nontarget mRNAs (f), and in HuR target and nontarget mRNAs (G). *p*‐values were calculated using the Kolmogorov–Smirnov test (KS) test

Next, we investigated whether m6A modification affects mRNA abundance in general using a young versus old cohort comparison. We first compared the relationship between an RNA abundance and degree of m6A methylation by plotting FPKM of m6A‐modified RNA fragments on the X‐axis and total RNA on the Y‐axis within the same cohort group (Figure 2a,b). Our results have shown that there is no correlation between degree of methylation and abundance of transcripts. We then analyzed the cumulative distribution fraction (CDF) of log2 fold change in RNA expression by comparing m6A‐methylated RNA with the rest between young and old cohort groups. Our results show that methylated RNAs tend to be more abundant than nonmethylated RNAs (Figure 3a–e) in old vs. young. In addition, CDF analysis of AUF1 and HuR target mRNAs (Mukherjee et al., 2011; Yoon et al., 2014) from MeRIP‐seq and total RNA‐seq data has shown that AUF1 and HuR target RNAs are downregulated further than nontarget mRNAs once they are methylated (Figure 3f,g, Figures S2 and S3). These results imply possible involvement of AUF1 and HuR in the stability regulation of m6A‐modified RNA (Visvanathan et al., 2017).

We also analyzed m6A‐modified RNA sequences adjacent to the translation start and stop codons of mRNA, and then identified that the preferential region of m6A modification on mRNAs is similar to each other (Figure S1). This observation was unexpected when we considered the notion that the highly uneven distribution of m6A modification along transcripts observed under the different cellular contexts that affect activity or presence of methyltransferases, m6A‐binding proteins, and demethylases. Together, these findings indicate that the quantity of RNA methylation, although not the type and region of methylation, fluctuates during aging.

### 2.2 AGO2 mRNA methylation is decreased in old PBMCs with changes in its steady‐state abundance

**Figure 4:**
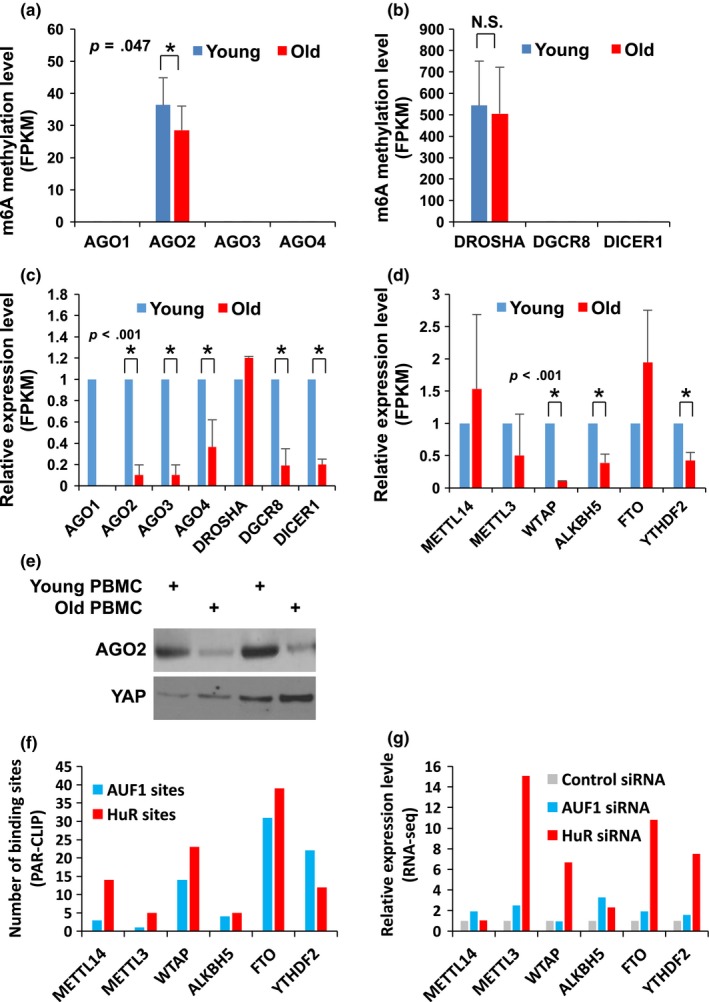
*AGO2* and *DROSHA* mRNAs are methylated in PBMCs. (a, b) Number of methylated fragments (FPKM) on *DROSHA* and *AGO2* mRNAs in young and old PBMCs. Data in A are statistically significant (*p *=* *.047, Student's *t*‐test) (c) Relative levels of *AGO1‐4, DROSHA, DGCR8,* and *DICER1* mRNAs in young and old PBMCs. (d) Relative abundance of *METTL14*,*METTL3*,*WTAP*,*ALKBH5*,*FTO*, and *YTHDF2* mRNAs in young and old PBMCs. All *p*‐values were calculated using Student's *t*‐test. (e) AGO2 protein level analyzed by Western blot in young and old PBMCs. (f) Number of AUF1 and HuR binding sites on the indicated mRNAs from PAR‐CLIP analysis (Yoon et al., 2014). (g) Relative expression level (FPKM) of the indicated mRNAs from total RNA‐seq after depletion of either AUF1 or HuR

Among many methylated mRNAs, we tried to find a key transcript in which differential methylation status modulates mRNA stability during the process of aging. We found that the mRNA encoding DROSHA, a ribonuclease that processes primary microRNAs, had a relatively higher methylation status than AGO2, a catalytic protein in miRNA‐mediated gene silencing, in both young and old PMBCs. Interestingly, *DROSHA* mRNA was highly methylated both in young and old PBMCs with no significant change, while methylation of *AGO2* mRNA decreased in the old PBMCs (Figure 4a,b, *p *= .047). The total RNA transcriptome of PBMCs revealed that *AGO2* mRNA is underexpressed in old PBMCs while the levels of *DROSHA* mRNA did not change significantly (Figure 4c and Table S2). This indicates that methylation on *AGO2* mRNA is implicated in mRNA stability, but *DROSHA* mRNA methylation may affect different stages of mRNA metabolism such mRNA maturation and translation. Interestingly, the mRNA encoding DICER1, a ribonuclease that processes primary microRNAs, was decreased in the old PBMCs possibly via an RNA decay factor, AUF1 as observed previously (Noren Hooten et al., 2016) had no detectable methylation both in young and old PBMCs. These results implicate that cells utilize various mechanisms to regulate mRNA stability for fine‐tuning regulation of gene expression in a given context.

When we compared mRNA expression involved in m6A's RNA modification pathway, we identified that steady‐state levels of *WTAP* mRNA, which encodes a regulatory subunit of the methyltransferase complex decreases dramatically while other mRNAs encoding methyltransferases, demethylases, and m6A‐binding proteins change in their abundance moderately in old PBMCs compared with young (Figure 4d). We also observed concomitant decrease in AGO2 protein level in old PBMCs when compared to young cohorts (Figure 4e). Although we still do not know how the expression level of m6A‐methyltransferases and demethylases fluctuated in PBMCs, we observed that there are several binding sites recognized by AUF1 or HuR on mRNAs encoding methyltransferases and demethylases. This result implies that the expression of methyltransferases and demethylases is regulated by AUF1‐ or HuR‐mediated mRNA decay or translation (Figure 4f,g). These findings also suggest that m6A modification of *AGO2* mRNA may stabilize it in young PBMCs, possibly due to expression change in methyltransferases and demethylases.

### 2.3 m6A modification stabilizes AGO2 mRNA in HDFs

**Figure 5:**
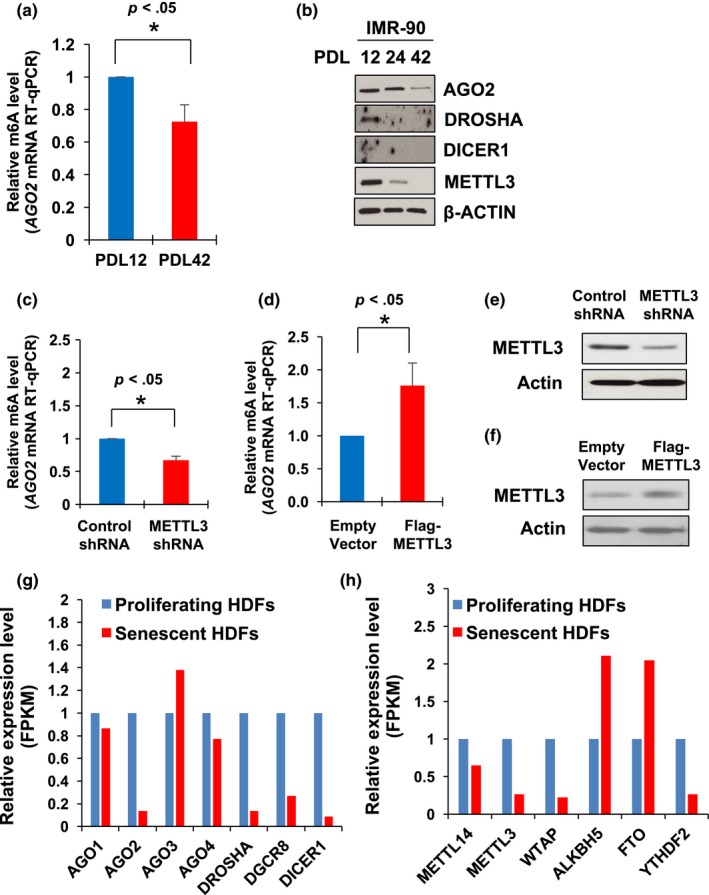
*AGO2* mRNA methylation correlates with AGO2 protein expression in HDFs. (a) Relative *AGO2* mRNA methylation between early and late passage IMR‐90 cells. (b) Expression level of indicated proteins is measured by Western blot analysis in early, middle, and late passage IMR‐90 cells (c and d) Depletion of METTL3 decreases abundance of m6A‐modified *AGO2* mRNA by comparing amount of *AGO2* mRNA from m6A immunoprecipitation. Overexpression of METTL3 induces *AGO2* mRNA methylation. (e and f) Efficiency of METTL3 depletion or overexpression was investigated by Western blots of whole‐cell lysates. (G and H) Relative abundance (FPKM) of *AGO1‐4, DROSHA, DGCR8, DICER1, METTL14*,*METTL3*,*WTAP*,*ALKBH5*,*FTO*, and *YTHDF2* mRNAs in proliferating and senescent HDFs. *p <* *.05 (Student's *t*‐test), compared with control condition

As cellular senescence is thought to be one of the driving factors contributing to human aging (Baker et al., 2011, 2016), we next investigated the change in *AGO2* mRNA methylation in HDFs under a different population doubling level (PDL). We observed a decline of m6A modification in late passage IMR‐90 (PDL‐42) similar to observations in human PBMCs (Figure 5a). Indeed, expression of AGO2 and METTL3 declined in late passage HDFs along with downregulation of DROSHA and DICER1 protein (Figure 5b), although decreased DROSHA and DICER1 expressions were not affected by m6A methylation in PBMCs (Figure 4b,c). Depletion of m6A methyltransferase METTL3 decreased *AGO2* mRNA methylation while METTL3 overexpression increased *AGO2* mRNA methylation (Figure 5c–f). However, METTL3 maybe not implicated in the regulation of *DROSHA* mRNA methylation (Figure S4a,b). Decrease in *AGO2* mRNA methylation correlates with decrease in *AGO2* mRNA level in senescent HDFs (Figure 5a,g), but there is a discrepancy on expression change in *AGO3* and *DROSHA* mRNAs in PBMCs (Figure 4c) and HDFs (Figure 5g). This might be due to an intrinsic property of fibroblasts and lymphocytes that could result in differential expression of mRNAs and proteins involved in senescence and aging, respectively. In HDFs, decline of *AGO2* mRNA methylation possibly originates from decreased expression of methyltransferases (Figure 5h). These observations suggest that m6A methylation increases expression of AGO2 and implicate this is due to a change in *AGO2* mRNA stability.

**Figure 6:**
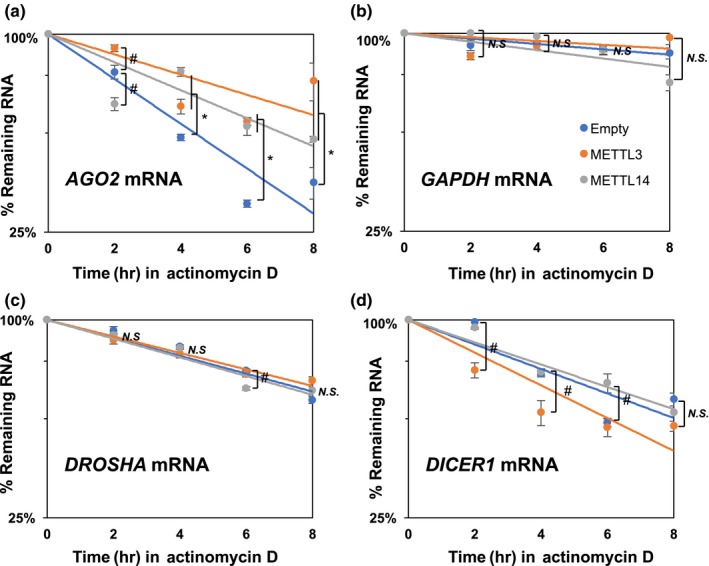
Stability of *AGO2*,*GAPDH*,*DROSHA*, and *DICER1* mRNAs in HDFs (a–d) Half‐life of *AGO2*,*GAPDH*,*DROSHA*, and *DICER1* mRNAs were measured in IMR‐90 after treatment of actinomycin D and comparison of remaining mRNAs by RT‐qPCR. * *p *<* *.05 (Empty vector vs. METTL3/METTL14 overexpression), # *p *< .05 (Empty vector vs. either METTL3 or METTL14 overexpression), *N.S*. (Not Significant)

The possibility of m6A‐mediated regulation of *AGO2* mRNA stability was investigated by overexpressing the components of the methyltransferase complex, such as METTL3 and METTL14, then measuring the amounts of remaining mRNAs once actinomycin D was used to treat IMR‐90 (PDL‐12) (Figure 6). Interestingly, overexpression of two major methyltransferases increases the half‐life (t_1/2,_ 50% of original abundance) of *AGO2* mRNA stability from 4.9 hours to 9.5 hours and 7.1 hours, respectively, but not *DROSHA*,* DICER1,* and *GAPDH* mRNA did not show a drastic change, which supports our observation (Figure 4a,b,c) that m6A methylation differentially regulates its target mRNA's fate, but the mechanisms are unknown. We also observed increase in *AGO2* mRNA stability after overexpression of METTL3 in IMR‐90 (PDL‐42) (Data not shown). This finding suggests that m6A modification on *AGO2* mRNA stabilizes it in HDFs. A possible explanation is that not all m6A methylation elicits the same consequence on mRNA stability.

### 2.4 Differential expression of mature miRNAs in young and old PBMCs

**Figure 7:**
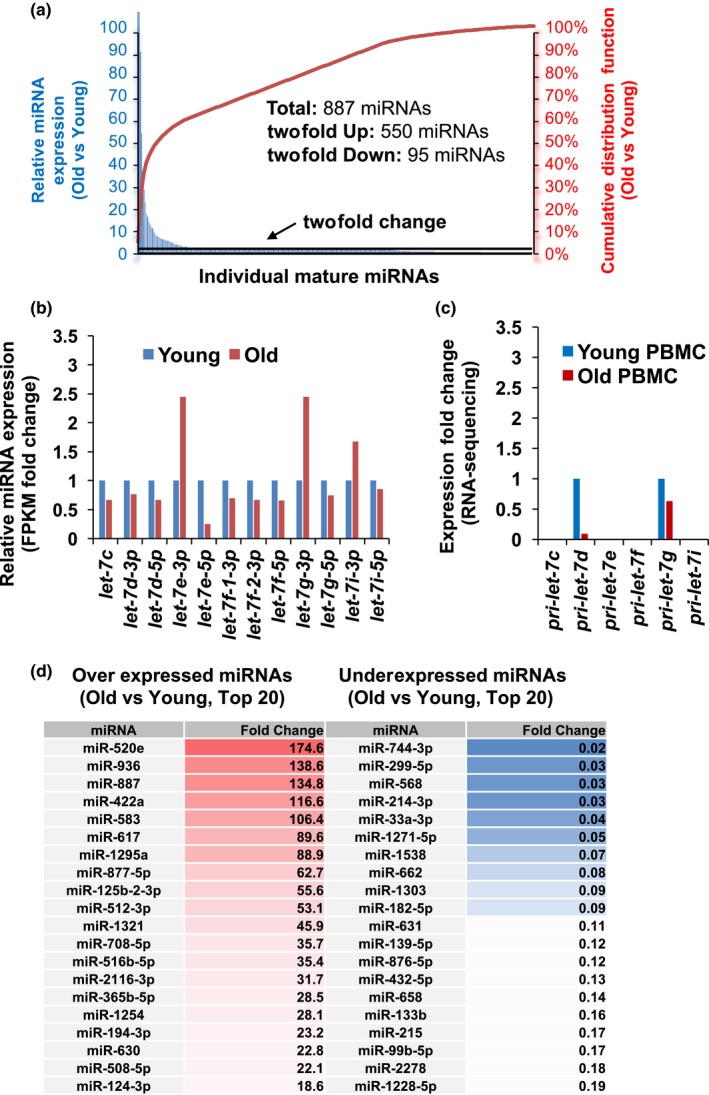
Changes of mature miRNA expression in young and old PBMCs. (a) Fold change of miRNAs in young and old PBMCs (y‐axis in blue on left) and cumulative distribution fraction of miRNAs in PBMCs (y‐axis in red on right) (b) Representative expression of let‐7 family miRNAs in young and old PBMCs. (c) Representative expression of primary let‐7 family miRNAs in young and old PBMCs from total RNA‐seq. (d) List of Top 20 miRNAs overexpressed or underexpressed in old PBMCs

Due to decreases in expression levels of *AGO2* mRNA in old PBMCs implicate a change in miRNA abundance as observed in mouse embryonic fibroblasts lacking AGO2 (Zamudio Jesse, Kelly Timothy & Sharp Phillip, 2014). In order to test whether the reduction of AGO2 expression due to differences in m6A modification leads to the reduction of mature miRNA level during aging, we performed miRNA microarray analysis of the young and old PBMCs. Our analysis revealed that among 887 detectable miRNAs, 550 miRNAs are overexpressed while 93 miRNAs are underexpressed (Figure 7a,d, and Table S3) showing the fluctuation of miRNA expression in aging process. When we analyzed *let‐7* family miRNAs whose level decreased in senescent fibroblasts (Marasa et al., 2010), we found that seven of 12 detectable *let‐7* miRNAs are downregulated in old PBMCs (Figure 7b). To determine whether fluctuation of mature miRNA level is due to change in miRNA processing, we compared expression level of primary *let‐7* family miRNA from total RNA‐seq data and observed that among detectable primary miRNAs, parental transcripts of *let‐7d* and *let‐7 g* decreased in old PBMCs (Figure 7c). Our results implicate the combinatorial contribution of miRNA transcription, processing, and decay on the abundance of mature miRNAs in young and old PBMCs.

**Figure 8:**
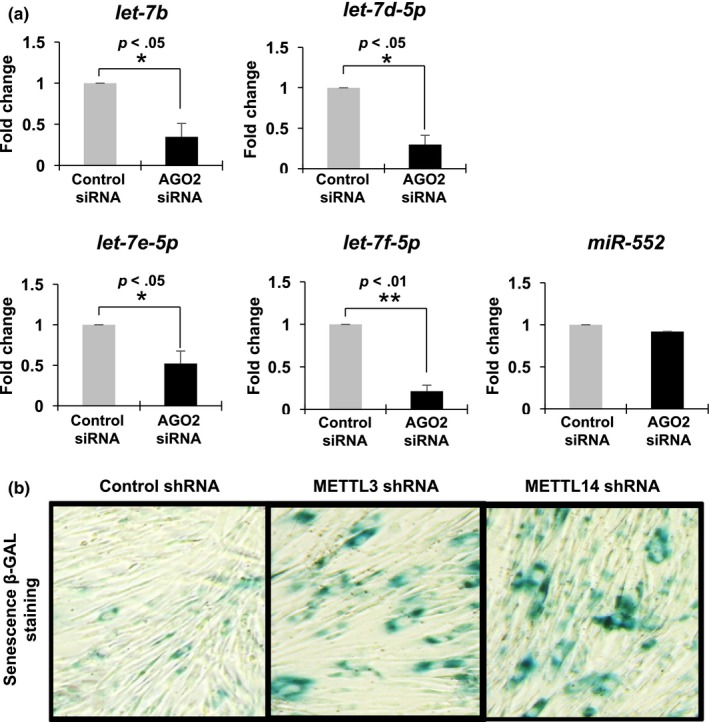
AGO2 is involved in regulation of subset of *let‐7* miRNAs expression and cellular senescence. (a) Relative steady‐state level of miRNAs after AGO2 depletion in early passage IMR‐90. * *p *<* *.05, ** *p *<* *.01 (Control siRNA vs. AGO2 siRNA) (b) Degree of cellular senescence is measured by beta‐galactosidase staining

Next, we examined if the level of *let‐7* family miRNAs are regulated by AGO2 in HDFs similar to PBMCs. To this end, we depleted AGO2 in early passage IMR‐90 cells, and then measured the steady‐state level of *let‐7* family miRNAs underexpressed in old PBMCs compared with young. Our RT‐qPCR analysis revealed that *let‐7b*,* let‐7d‐5p*,* let‐7e‐5p,* and *let‐7f‐5p* were decreased following knockdown of AGO2 in IMR‐90 cells (Figure 8a), but not *miR‐552* as an example of nonaffected miRNA. AGO2‐mediated maintenance of aging‐associated miRNA level is related to senescence phenotype as we observed that activity of senescence‐associated β‐galactosidases increased after depletion of either METTL3 or METTL14 (Figure 8b). These results demonstrate that m6A modification on *AGO2* mRNAs contributes to cellular senescence by regulating expression of mature miRNAs.

## 3 DISCUSSION

Our study reveals an m6A methylation profile of human aging, regulation of *AGO2* mRNA stability via its methylation, and changes in miRNA profiles of PBMCs. Our findings above suggest that 1) miRNA abundance is regulated by *AGO2* mRNA stability modulation, 2) the stabilization of *AGO2* mRNA is methylation‐dependent, and 3) *AGO2* mRNA decay contributes to senescence and aging.

If the expression levels of DROSHA, DGCR8, and DICER1 are crucial for miRNA abundance in cellular senescence and aging, then we should observe that almost all miRNAs are underexpressed due to the depletion of DROSHA, DGCR8, and DICER1 in senescent HDFs and old PBMCs. However, senescent HDFs exhibit a subset of miRNAs that are either overexpressed or underexpressed relative to proliferating HDFs (Marasa et al., 2010). This suggests that there are other key factors that modulate specific miRNA abundance. AGO2 is an ideal protein to modulate miRNA abundance in cellular senescence and aging since its association with mature miRNAs prevents them from degradation without affecting canonical miRNA processing (Zamudio Jesse et al., 2014).

Since methylation is used to physically mark mRNA for regulation by decay, translation, or localization (Licht & Jantsch, 2016), utilizing methylation to determine mRNA fate for mammalian cells would be an excellent strategy. Similar to RNA–protein and RNA–RNA interaction, mRNA methylation is dynamic but reversible only by the covalent removal of the methyl group(s) from the modified mRNA. This process can be tightly regulated by localization, activity, specificity, and affinity of methyltransferases and demethylases that target mRNAs, this process is similar to post‐translational protein modification. The change in the level of m6A modifiers in old PBMCs and senescent HDFs implies their involvement in mammalian aging and cellular senescence (Figures 4d and 5h). Dynamic regulation of m6A modification and mRNA decay is also dictated by position‐specific m6A methylation within the mRNA, and presence/activity of m6A‐binding proteins within a given context (Alarcón et al., 2015; Wang & He, 2014; Wang et al., 2014, 2015). A possibility is that *DROSHA* mRNA methylation is maintained by the presence of m6A readers or m6A‐regulated factors such as HuR, FMR1, and G3BP, resulting in preservation of *DROSHA* mRNA levels during the aging process (Edupuganti et al., 2017; Visvanathan et al., 2017). This may explain why *DROSHA* mRNA levels did not change despite having higher methylation levels compared with *AGO2* mRNA. Further investigation will determine if there is a differential composition of the RNP complex on methylated *AGO2* and *DROSHA* mRNA that regulates their decay regulation during aging. Also, how the signaling pathways and cellular stresses influence differential distribution patterns of RNA methylation is not yet well defined. In particular, the factors that are attributable to changes in m6A modification of some mRNA but not all mRNAs during aging need to be explored in the future.

While depletion of DGCR8, and DICER1 induces spontaneous senescence (Gómez‐Cabello, Adrados & Palmero, 2013; Mudhasani et al., 2008), AGO2 depletion itself did not promote cellular senescence but sensitized it to RAS‐oncogene‐induced cellular senescence (Yang et al., 2014). These findings suggest that AGO2 mRNA demethylation and subsequent decrease in AGO2 level are not sufficient but could be required for cellular senescence by METTL3 or METTL14 depletion (Figure 8b). This difference is critical for modulating cellular senescence as it is generally considered to be an irreversible process. Even when reintroducing miRNA processing enzymes, it is difficult to reverse cellular senescence, but the re‐expression of AGO2, with tight modulators of its abundance, will attenuate senescence. In addition, recovering the expression of *let‐7* should have similar results to recovering AGO2 because they both repress the expression of similar target genes. We observed a fluctuation in miRNA level during the aging process (Figure 7a,d). Although a subset of *let‐7* family miRNAs are downregulated, the rest are not affected in their abundance. Differential expression of *let‐7* family miRNAs may arise from the existence of novel‐miRNA‐binding proteins such as AUF1 and HuR possibly affecting the stability of mature miRNAs (Yoon, Abdelmohsen, Kim, et al., 2013; Yoon et al., 2015). Thus, miRNA abundance could be influenced by other RNA‐binding proteins whose expression and/or activity depends on a cellular context. The identification of mRNAs targeted by *let‐7* family miRNAs in senescence and aging should be a goal of future studies. These mRNAs include *IGF1R*,* INSR*,* IRS2*,* HMGA2*, and *IGFBP2* (Zhu et al., 2011) which are essential for energy metabolism, a key aspect of cellular senescence. A recent study analyzed a large number of blood samples from Framingham Heart Study (FHS) participants and investigated if miRNA expression is related to chronological age in FHS participants (Huan et al., 2017). They observed most miRNA were underexpressed in old individuals; however, *let‐7* family members were not found as age‐associated miRNAs in their study, possibly due to a large sample size with a wide age range compared with our study.

Taken together, our observations demonstrated mechanisms that regulate miRNA expression via methylation‐dependent *AGO2* mRNA abundance in cellular senescence and aging. Future mechanistic studies will reveal how *AGO2* mRNA methylation represses decapping and deadenylation and how AGO2 stabilizes mature miRNA from 5'‐to‐3' decay and uridylation‐mediated decay.

## 4 METHODS

### 4.1 Human study participants

For RNA profiling, a subcohort of young (30 years) and old (64 years) participants from the “Healthy Aging in Neighborhoods of Diversity across the Life Span study” (HANDLS) (Evans et al., 2010; Noren Hooten et al., 2016) was chosen for examination of m6A modification, total transcriptome, and miRNA microarray by age. Clinical information on this subcohort has been described previously and is also listed in Figure 1a (*n *= 11/group) (Noren Hooten et al., 2010, 2016). The HANDLS study is approved by the Institutional Review Board of the National Institute of Environmental Health Sciences, National Institutes of Health. All of the participants signed a written informed consent document.

### 4.2 Cell culture, transfection, and plasmids

Human IMR‐90 cells (PDL‐12) were cultured in DMEM (Invitrogen) supplemented with 10% (v/v) FBS and antibiotics. Plasmids were transfected at 1–2 μg/ml [pcDNA3, METTL3, METTL14 from Addgene]. Transfected cells were analyzed 48 h later after actinomycin D treatment (2.5 μg/ml) at different time periods as indicated. At each time point, total RNA was isolated using TRIzol according to the manufacturer's instructions, and used for RT‐qPCR analysis to determine RNA half‐life (t_1/2_), (the time needed for each transcript to reach 50% of their original abundance). Transcript levels were normalized to the abundance of 18S rRNA.

### 4.3 RT‐qPCR analysis

Total RNA was isolated, and cDNA was generated from 0.5μgs of RNA using random hexamers and reverse transcriptase (Maxima, Thermo Scientific). qPCR was carried out using SYBR green master mix (Kapa Biosystems), and a thermal cycler (Bio‐Rad). The primers used for RT‐qPCR are provided in Table S4. The relative quantities of mRNAs were calculated using the ΔΔCt method and normalized using human 18S as endogenous control.

### 4.4 High‐throughput RNA analysis

We analyzed global m6A modification, transcriptome, and microRNA expression from human participants as follows. We first isolated total RNA from the human study participants using TRIzol (Invitrogen). For m6A profiling, 1 ug of total RNA was fragmented using Bioruptor for 30 min, resulting in 100–200 nt RNA fragments. Immunoprecipitation of small RNAs with an m6A‐specific antibody (from Synaptic Systems) enriched the amount of methylated RNAs in the immunopellets. Once m6A RNAs are recovered, small RNA library preparation with the Illumina small RNA library preparation kit (Illumina GAII). The libraries were run in GAII, and the resulting data were aligned to human genome sequences (HG19) for quantitation and motif determination. For transcriptome analysis, we followed the standard protocol of Ion Torrent Proton sequencing. For microRNA profiling, Agilent human miRNA Microarray 15.0. was performed and individual miRNAs with pairwise z‐test *p* value <.05, absolute value of Z ratio >1.5, and with FDR <0.3 for significant change.

### 4.5 Western blot analysis

Whole‐cell lysates, prepared in radioimmunoprecipitation assay (RIPA) buffer, were separated by sodium dodecyl sulfate‐polyacrylamide gel electrophoresis (SDS‐PAGE) and transferred onto nitrocellulose membranes (Invitrogen iBlot Stack). Primary antibodies recognizing DROSHA, DICER1, METTL3, and Actin were purchased from Cell Signaling Technology. AGO2 antibody was purchased from Abcam. Horse Radish Peroxidase (HRP)‐conjugated secondary antibodies were purchased from GE Healthcare.

### 4.6 MeRIP‐qPCR analysis

Total RNA was isolated from IMR‐90 cells using TRIzol according to manufacturer's instructions. 5 μg of RNA was incubated with m6A‐specific antibody (from Synaptic Systems, 202 003) for 3 hr. The complexes were incubated with protein A‐sepharose beads for 2 hr at 4^◦^C. After the beads were washed with NT2 buffer (50 mmol/L Tris–HCl at pH 7.5, 150 mmol/L NaCl, 1 mmol/L MgCl2, and 0.05% NP‐40), bead‐associated RNA was extracted with acidic phenol. Isolated RNA was further assessed by reverse transcription (RT)‐quantitative polymerase chain reaction (qPCR) analysis using *AGO2* mRNA primers (Table S4). 18S rRNA primers (Table S4) were used as a nonspecific binding control.

### 4.7 Cumulative distribution fraction analysis

Cumulative distribution analysis of mRNA log_2_ fold changes expression was performed in R. For each group of interest, the empirical cumulative distribution functions of log_2_ fold mRNA expression values were computed using the ecdf function, and pairs of curves to be compared were displayed with plot.cdf. Significance of difference between the cumulative distribution curves was determined using the KS (Kolmogorov–Smirnov) test, and the corresponding *p*‐value was included in the plot.
