# Supplementary figures and images for: Profiling of m6A RNA modifications identified an age‐associated regulation of AGO2 mRNA stability
Source: Aging Cell. 2018 Mar 23;17(3):e12753. doi: 10.1111/acel.12753 (PMC5946072; doi:10.1111/acel.12753)

**A**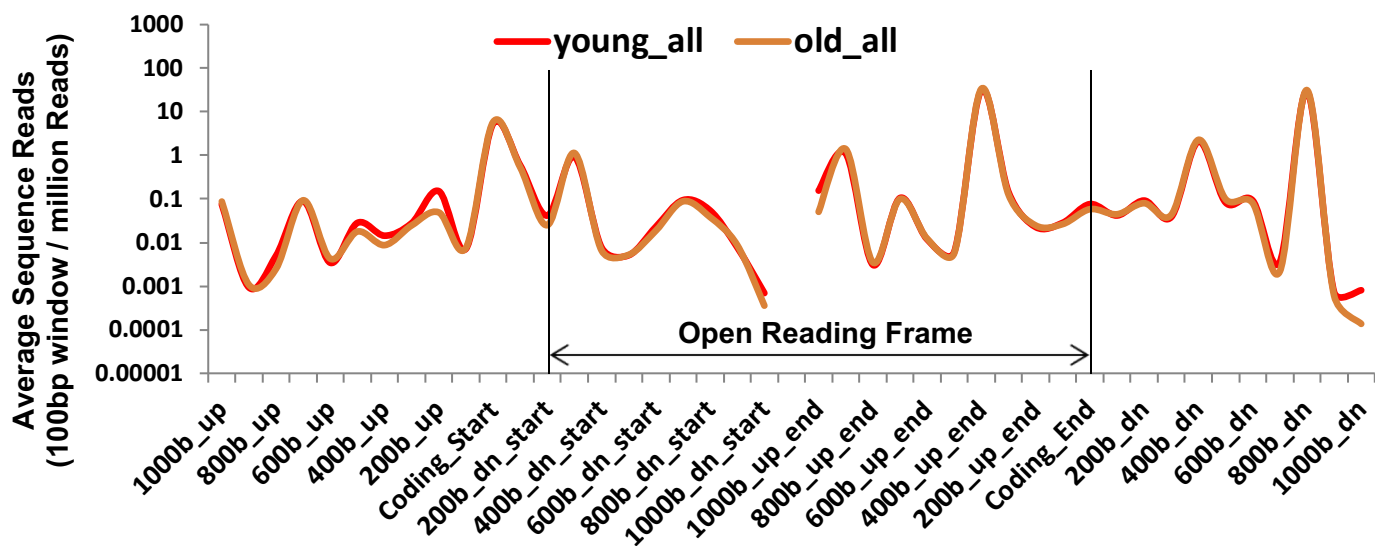**B**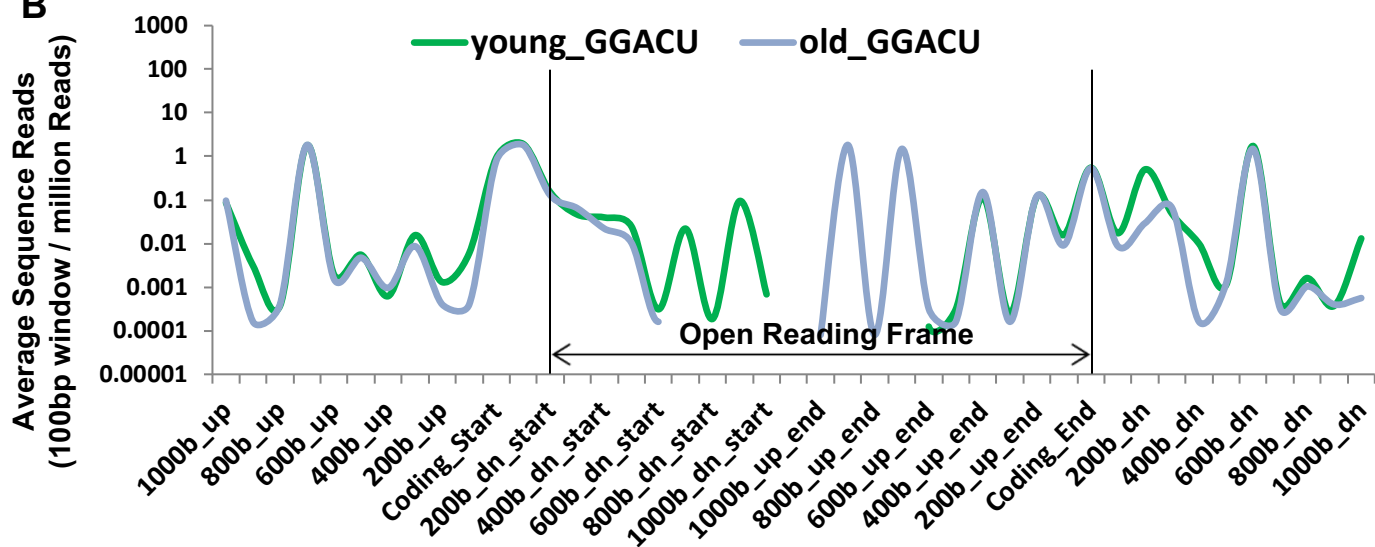

**C**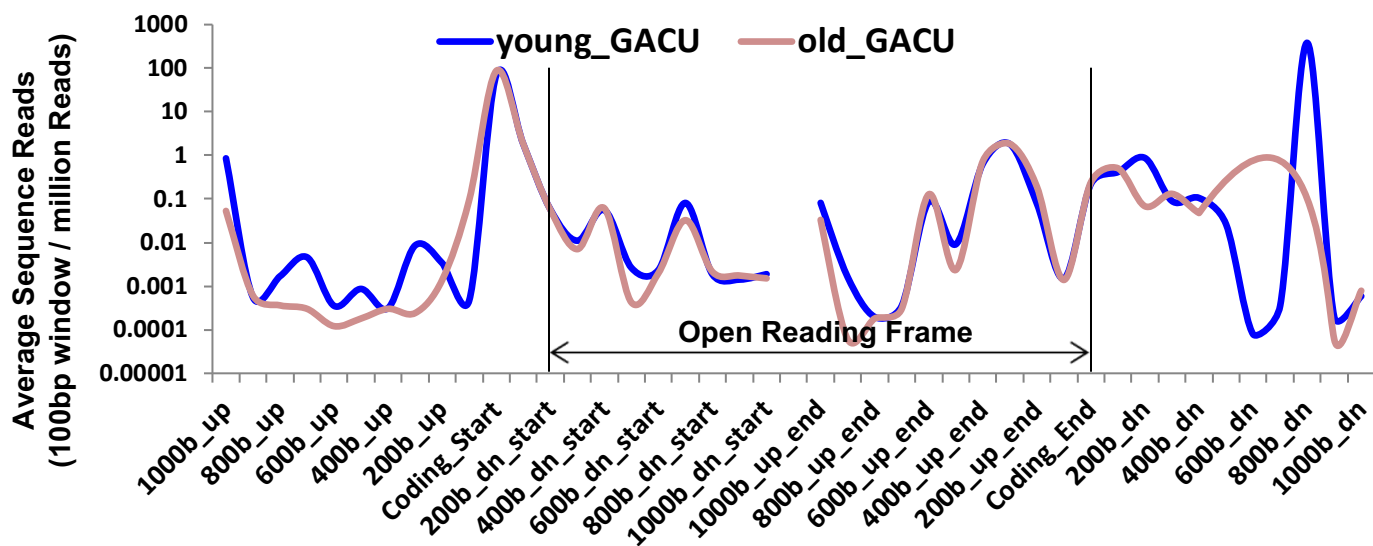**D**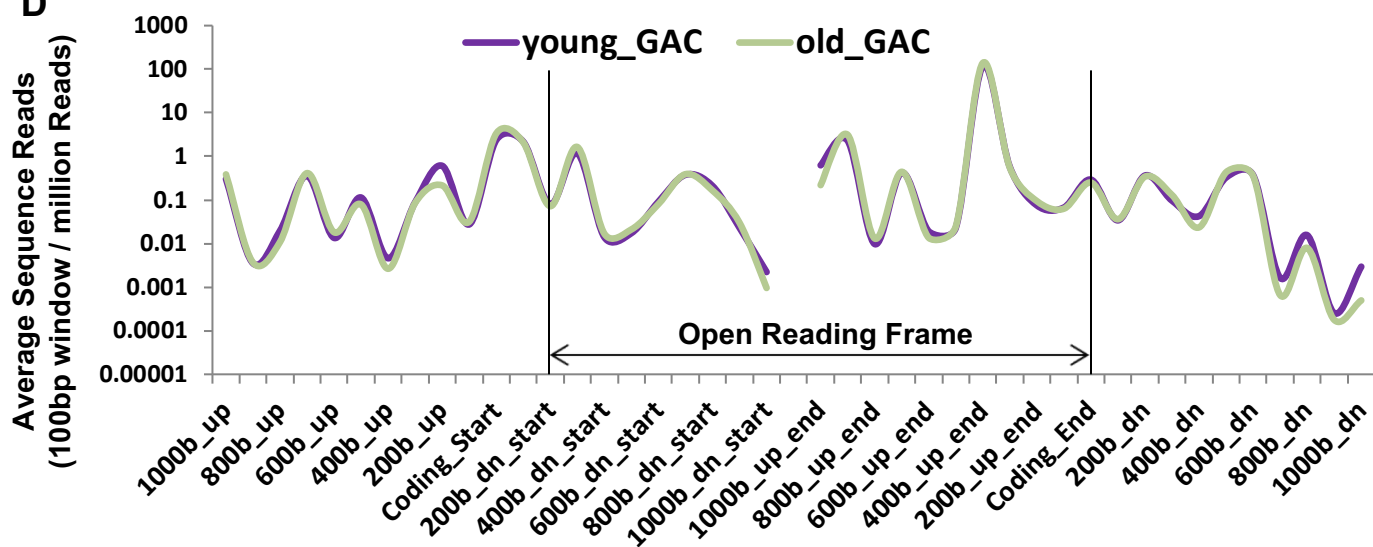**E**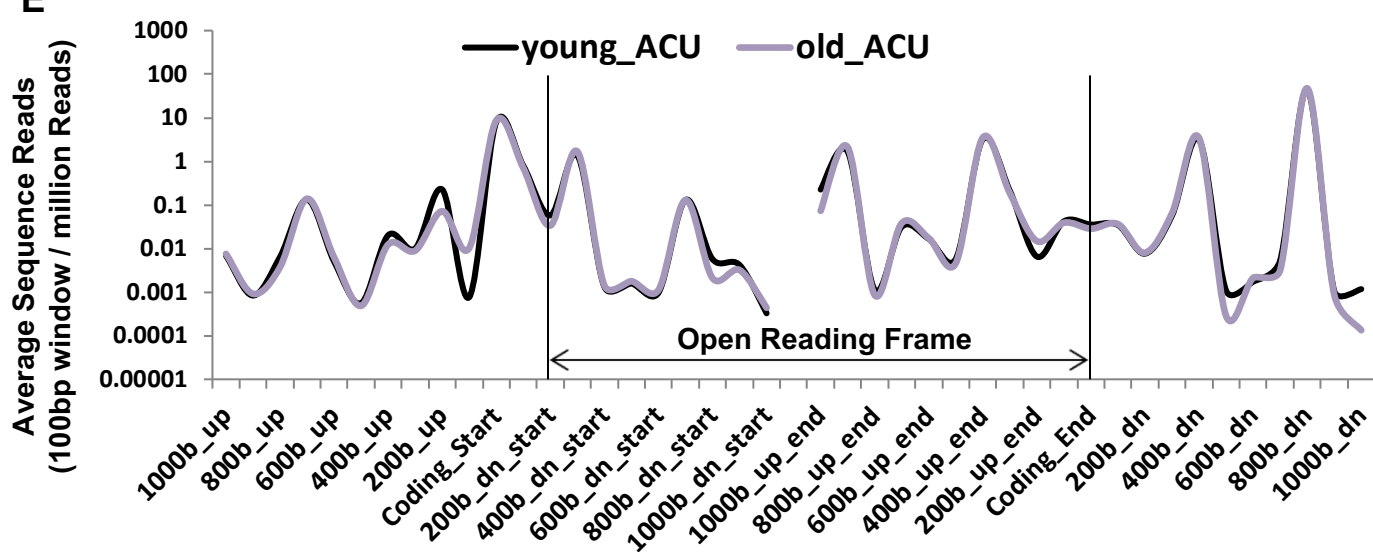

**A**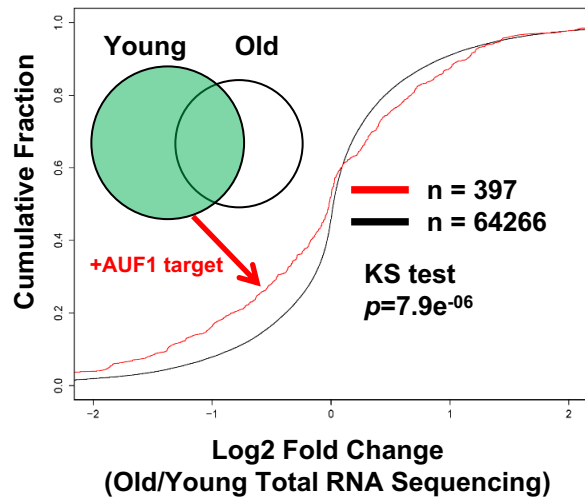**B**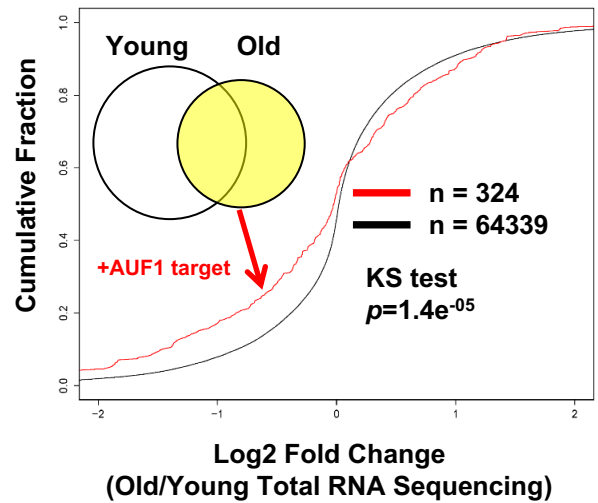**C**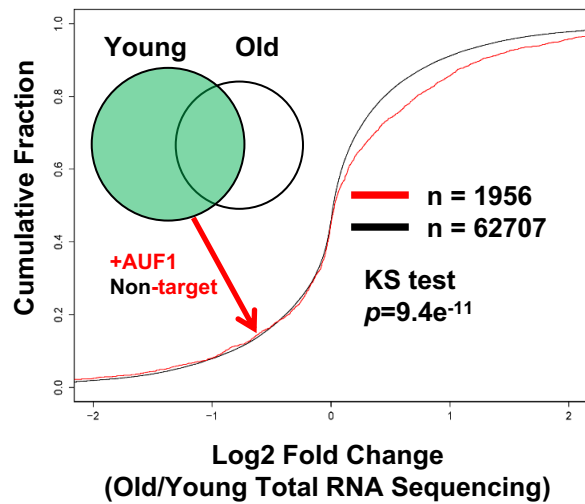**D**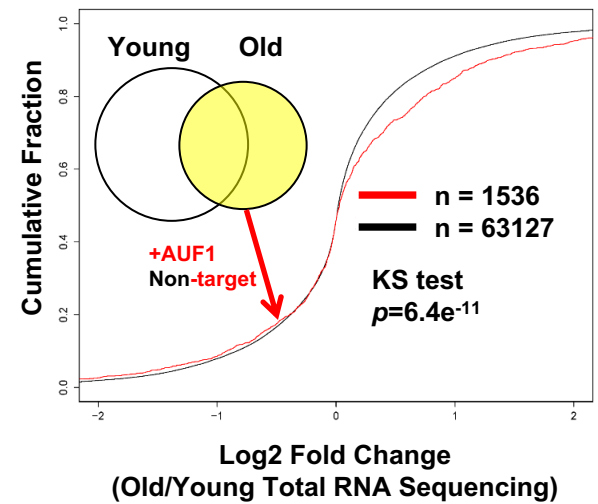

**A**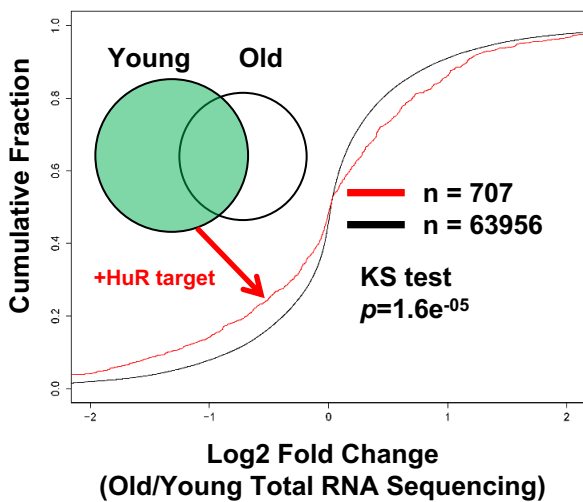**B**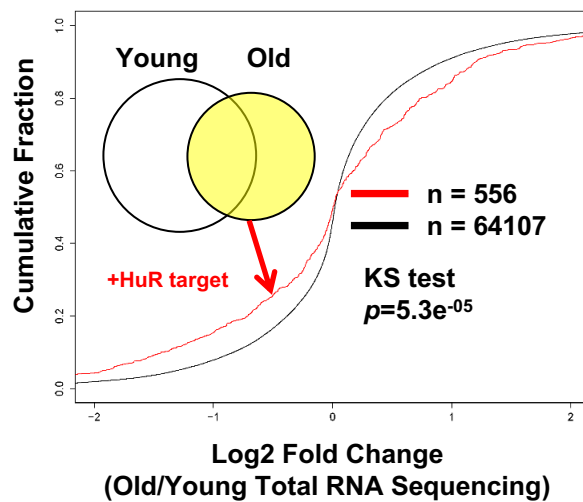**C**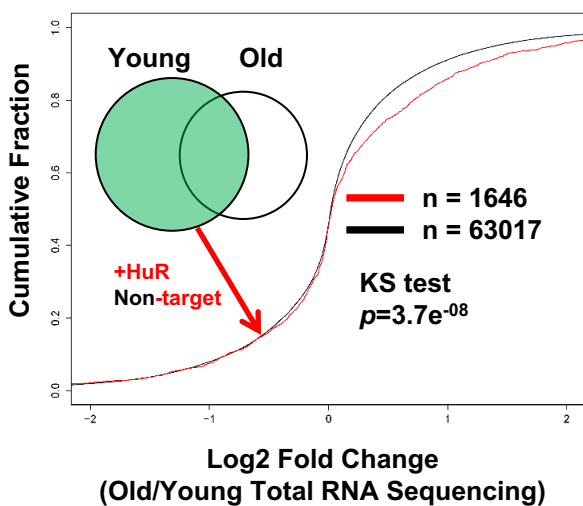**D**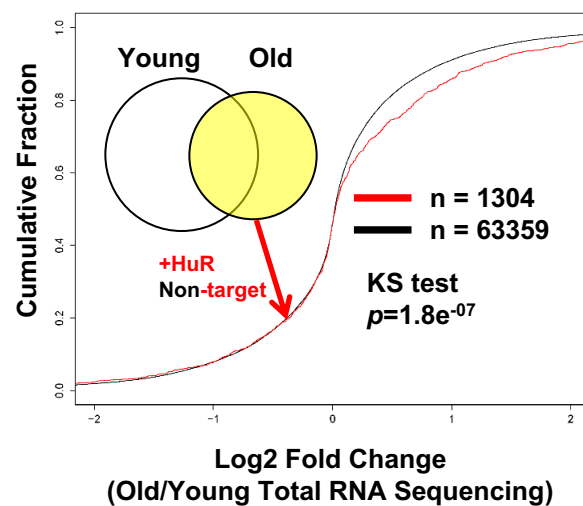

**A**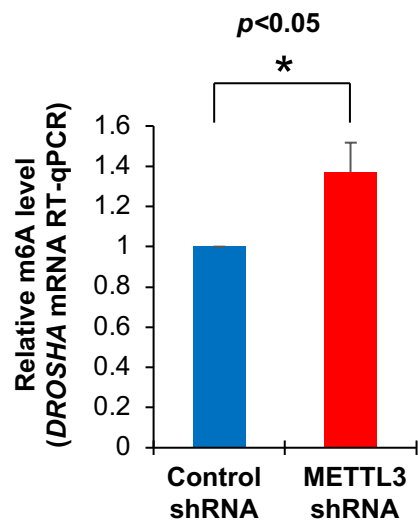**B**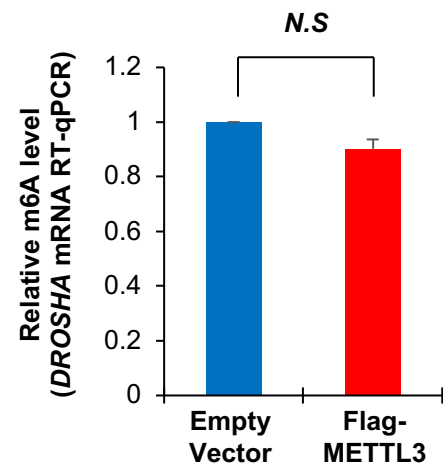

Supplement: Supplementary file 1 [file ACEL-17-e12753-s001.pdf]
