# Supplementary material for: Profiling of m6A RNA modifications identified an age‐associated regulation of AGO2 mRNA stability
Source: Aging Cell. 2018 Mar 23;17(3):e12753. doi: 10.1111/acel.12753 (PMC5946072; doi:10.1111/acel.12753)
Supplement: Supplementary file 6 [file ACEL-17-e12753-s006.pdf]

Profile of m6A RNA modification identified age-associated regulation of *AGO2* mRNA stability.

Kyung-Won Min[1,ǂ], Richard W. Zealy[1,ǂ], Sylvia Davila[1,ǂ], Mikhail Fomin[1], James C. Cummings[1], Daniel Makowsky[1], Catherine H. Mcdowell[1], Haley Thigpen[1], Markus Hafner[2], Sang-Ho Kwon[3], Constantin Georgescu[4], Jonathan D. Wren[4] and Je-Hyun Yoon[1,5,] *

1 Department of Biochemistry and Molecular Biology, Medical University of South Carolina, Charleston, SC 29425, USA

2 Laboratory of Muscle Stem Cells and Gene Regulation, National Institute of Arthritis and Musculoskeletal and Skin Diseases, 50 South Drive, Bethesda, MD 20892, USA.

3 Department of Medicine, Division of Nephrology, Medical University of South Carolina, Charleston, SC 29425

4 Arthritis and Clinical Immunology Research Program, Division of Genomics and Data Sciences, Oklahoma Medical Research Foundation; 825 N.E. 13th Street, Oklahoma City, OK 73104-5005, USA

5 Laboratory of Genetics, National Institute on Aging-Intramural Research Program, NIH, Baltimore, MD 21224, USA

[*]Correspondence:
Medical University of South Carolina
173 Ashley Avenue
Charleston, SC 29425, USA
Tel: 843-792-9318; Fax: 843-792-8304
yoonje@musc.edu
Jonathan-Wren@omrf.org

ǂ **These authors contributed equally in this work**

Supplemental Figure S1. m6A profile over coding regions in PBMCs. (A-E) Positions of m6A RNA fragments from translation start and stop codons. Data sets from RNA sequencing were binned with all motifs *(A)*, GGACU *(B)*, GACU *(C)*, GAC *(D)*, and ACU (*E)* in young and old PBMCs.

Supplemental Figure S2. **AUF1 regulates abundance of m6A-methylated target mRNAs. (A-D)** Cumulative distribution analysis of log2 fold changes in m6A-modified AUF1 target and non-target mRNA expression in young and old cohorts.

Supplemental Figure S3. HuR regulates abundance of m6A-methylated target mRNAs. (A-D) Cumulative distribution analysis of log2 fold changes in m6A-modified HuR target and non-target mRNA expression in young and old cohorts.

Supplemental Figure S4. METTL3 is not required for *DROSHA* mRNA methylation. (**A, B**) Depletion of METTL3 increases abundance of m6A-modified *DROSHA* mRNA slightly by comparing amount of *DROSHA* mRNA from m6A immunoprecipitation. Overexpression of METTL3 did not change *DROSHA* mRNA methylation.

Table S1. RNA fragments of m6A modification in young and old PBMCs
Table S2. Total RNA sequencing in young and old PBMCs
Table S3. miRNA microarray in young and old PBMCs
Table S4. Primers used in this study
